# Managing stigma and reconstructing caregiver identity: a qualitative descriptive study of family caregivers of children with cerebral palsy in China

**DOI:** 10.3389/fpsyg.2026.1933578

**Published:** 2026-09-15

**Authors:** Mei Yu, Cuiping Xu, Chunmin Ji, Zhen Zhang, Meiying Guo

**Affiliations:** 1 School of Nursing, Shandong First Medical University & Shandong Provincial Academy of Medical Sciences, Jinan, China; 2 Hospital Vice President’s Office, The First Affiliated Hospital of Shandong First Medical University & Shandong Provincial Academy of Medical Sciences, Jinan, China; 3 The Second Affiliated Hospital of Shandong First Medical University & Shandong Provincial Academy of Medical Sciences, Taian, China; 4 Department of Radiation Oncology, Qilu Hospital of Shandong University, Jinan, China

**Keywords:** caregiver identity, cerebral palsy, family caregivers, qualitative research, stigma management

## Abstract

**Purpose:**

Family caregivers of children with cerebral palsy may encounter stigma alongside substantial caregiving demands. However, less is known about how they experience and manage stigma in everyday social encounters and reconstruct a valued caregiver identity. This study aimed to explore these experiences among family caregivers of children with cerebral palsy in China.

**Methods:**

A qualitative descriptive design was used. Twenty family caregivers were recruited from a tertiary hospital in China using purposive sampling. Data were collected through semi-structured, one-to-one interviews and analysed using inductive content analysis.

**Results:**

Twenty family caregivers participated in the study. Analysis generated four themes: (1) becoming a caregiver: disrupted expectations and expanded family roles; (2) living under the gaze: stigma and social judgment in public and family encounters; (3) managing stigma in everyday interactions: concealment, disclosure, and resistance; and (4) rebuilding a valued caregiver identity through responsibility, support, and meaning-making. Caregivers experienced public/enacted stigma, stigma by association, anticipated stigma and, in some accounts, internalized affiliate stigma. They used context-dependent strategies to manage stigma, while continued caregiving, children’s small gains, and supportive relationships helped sustain a valued caregiver identity despite uncertainty about the future.

**Conclusion:**

Stigma-related experiences extended beyond internalized affiliate stigma and were embedded in everyday social relationships. Family-centered, stigma-sensitive care should address stigma-related challenges across family, community, and service contexts while strengthening support that affirms caregivers’ value and identity.

## 1 Introduction

Cerebral palsy is a group of permanent disorders of movement and posture caused by non-progressive disturbances in the developing brain, often accompanied by epilepsy, intellectual disability, communication difficulties, feeding problems, sensory impairments, and secondary musculoskeletal complications (Patel et al., 2020; Vitrikas et al., 2020). It is one of the most common causes of childhood motor disability (Sadowska et al., 2020; Panda et al., 2024). Recent global analyses suggest that the birth prevalence of cerebral palsy in high-income countries has declined to approximately 2∼3 per 1,000 live births (Patel et al., 2020; McIntyre et al., 2022; Paneth and Yeargin-Allsopp, 2022), whereas available data from low- and middle-income countries indicate a markedly higher prevalence, with some estimates two to three times higher than those reported in high-income settings (McIntyre et al., 2022; Jahan et al., 2024; Khandaker et al., 2019). Approximately 6 million people with cerebral palsy have been reported in China, where family caregivers have described exhaustion, restricted daily life, and uncertainty about the future (Ni et al., 2022). Because cerebral palsy is lifelong and has no curative treatment, many children require sustained rehabilitation, daily assistance, and long-term involvement of family caregivers across childhood and adolescence (Trabacca et al., 2016; Mwinbam et al., 2023; Al Imam et al., 2021).

The consequences of cerebral palsy therefore extend beyond the child and shape family caregiving over a prolonged period. Recent evidence has shown that families of children with cerebral palsy face not only medical and out-of-pocket expenses, but also substantial time demands related to daily care and rehabilitation (Nova-Díaz et al., 2025). In resource-limited settings, qualitative studies have further shown that caregiving difficulties are often compounded by limited information, loss of work, inadequate family and community support, social isolation, stigma, poverty, and insufficient public support (Vadivelan et al., 2020; Poojari et al., 2022; Seroke and Mkhize, 2023). These findings indicate that caregivers’ experiences are shaped not only by the practical demands of long-term rehabilitation and daily care, but also by social conditions such as stigma, limited support, and restricted participation. For children with disabilities, stigma can also contribute to social exclusion and restricted access to health and education, limiting their well-being and participation (Smythe et al., 2020). Affiliate stigma is one important dimension of this caregiving context. Affiliate stigma refers to the internalization of negative public attitudes by individuals closely associated with a stigmatized person, including parents and family caregivers (Shi et al., 2019; Becerra Carrillo et al., 2024). However, stigma-related experiences among family caregivers are not limited to affiliate stigma. Caregivers may also encounter public/enacted stigma, stigma by association when they themselves are devalued because of their relationship with the child, and anticipated stigma when they expect negative reactions from others. These related forms of stigma are conceptually distinct from internalized affiliate stigma (Dako-Gyeke et al., 2021). In cerebral palsy caregiving, such stigma-related experiences may occur when disability is misunderstood, morally judged, or attributed to parental failure (Dako-Gyeke et al., 2021). A Malaysian study of parents of individuals with cerebral palsy found that higher caregiver stress was associated with higher affiliate stigma, while lower stress was associated with better quality of life (Chu et al., 2023). It also highlighted the relevance of Asian cultural contexts, where social acceptance, family reputation, and public judgment may shape parents’ experiences of and responses to disability-related stigma (Chu et al., 2023). It is important to note that disability-related stigma may be closely tied to concerns about family reputation, loss of face, shame, and fear of social rejection, which may further shape how family caregivers perceive and respond to stigma-related social evaluation, in Chinese cultural contexts (Holroyd, 2003; Chiu et al., 2013; Huang et al., 2020; Ng and Ng, 2022).

Although existing research has established links between cerebral palsy caregiving, psychosocial burden, and affiliate stigma (Vadivelan et al., 2020; Chu et al., 2023), less is known about how family caregivers manage stigma in everyday social encounters and how these experiences shape their understanding of themselves as caregivers. In particular, limited attention has been paid to how caregivers experience and manage stigma-related encounters across public, family, school, and community contexts, and how they reconstruct a valued caregiver identity while living with caregiving demands, uncertainty, and social evaluation in the Chinese context. Therefore, this study aimed to explore how family caregivers of children with cerebral palsy in China experience and manage stigma-related social encounters and how they reconstruct a valued caregiver identity. By focusing on stigma management and caregiver identity reconstruction, this study seeks to extend current understanding beyond caregiver burden and psychological adjustment, and to inform stigma-sensitive, family-centered support in rehabilitation, nursing, education, and community settings.

## 2 Materials and methods

### 2.1 Study design

This study employed a qualitative descriptive design (Sandelowski, 2000). This approach was chosen to provide an in-depth, practice-relevant exploration of how family caregivers of children with cerebral palsy experience and manage stigma, and how they reconstruct a valued caregiver identity within everyday caregiving and rehabilitation contexts, rather than to generate a new theoretical or conceptual framework. No formal *a priori* theoretical or conceptual framework was used to guide the study. Qualitative description was appropriate because it supports the development of findings that remain close to participants’ accounts while being readily interpretable and applicable within clinical, rehabilitation, and family support contexts (Sandelowski, 2010; Doyle et al., 2020). The reporting of this study was guided by the Standards for Reporting Qualitative Research (O’Brien et al., 2014).

### 2.2 Study participants and selection procedures

Participants were recruited using purposive sampling from The First Affiliated Hospital of Shandong First Medical University, Jinan, China, which provides rehabilitation and follow-up services for children with cerebral palsy. Purposive sampling was used because the study sought information-rich accounts from caregivers who had direct experience of caregiving, rehabilitation accompaniment, social interaction, and stigma-related encounters in the context of childhood cerebral palsy (Robinson, 2014). Eligible participants were family caregivers who were aged 18 years or older, were the child’s main family caregiver or primary rehabilitation companion, had experience of caring for a child diagnosed with cerebral palsy, were able to describe their caregiving experiences, and provided informed consent (Tang et al., 2015). Paid caregivers and individuals who were unable to participate in an interview because of severe communication or cognitive difficulties were excluded.

The sample was relatively homogeneous in relation to the phenomenon under study, as all participants were family caregivers involved in the care or rehabilitation accompaniment of children with cerebral palsy. At the same time, maximum variation was sought to obtain rich accounts of stigma-related caregiving experiences and caregiver identity reconstruction (Patton, 2002). The research team therefore sought to include caregivers with different relationships to the child, durations of primary caregiving, places of residence, educational levels, and family economic situations, as well as children with different ages, sexes, and cerebral palsy types. Participant characteristics were reviewed iteratively during recruitment, and the research team purposively sought caregivers from less represented backgrounds to broaden variation across these characteristics.

Potential participants were initially identified with the assistance of healthcare professionals at the study site. Subsequently, eligibility was confirmed against the prespecified inclusion and exclusion criteria by two members of the research team with extensive experience in qualitative research (CX and MG). Following eligibility confirmation, MY contacted eligible caregivers either face-to-face or by telephone, explained the purpose and procedures of the study, provided study information, and invited them to participate voluntarily. Twenty-six caregivers were approached. Twenty-four initially agreed to participate, while two declined because of treatment-related commitments or other personal reasons. Four participants subsequently chose to withdraw during the interview process without providing a specific reason. These participants did not complete the formal interviews; although partial interview materials were collected, they were considered insufficient for analysis and were excluded from the final dataset. Twenty caregivers who completed the formal interviews were included in the final analysis. Recruitment and preliminary analysis proceeded iteratively. Methodological guidance for interview-based qualitative research suggests that sample size should be considered during study planning and ethics review, while remaining responsive to data richness and the developing analysis (Wutich et al., 2024). Previous empirical work also suggests that approximately 16–24 interviews may be sufficient to achieve meaning saturation in in-depth interview studies, although sample adequacy should remain responsive to the characteristics and information needs of the individual study (Hennink et al., 2017). Information sufficiency in the present study was assessed through ongoing comparison of successive interviews and discussion among MY, CJ, and ZZ. When three consecutive interviews generated no new codes, categories, or relevant insights related to the study aim, the research team considered that sufficient information had been obtained and recruitment was discontinued. In total, 20 family caregivers of children with cerebral palsy completed the study and were included in the final analysis.

### 2.3 Data collection

Data were collected between April 2025 and January 2026 through semi-structured, one-to-one interviews. Before each interview, basic demographic information about the caregivers and clinical information about the children were collected using a brief participant information form and medical records. Semi-structured interviews were used because they allow participants to describe experiences that are meaningful to them while enabling the interviewer to explore relevant issues in greater depth (Kallio et al., 2016).

The interview guide was developed based on the study aim, relevant literature, expert consultation, and discussion within the research team (Kallio et al., 2016). It included open-ended questions on caregiving role transitions, stigma-related encounters, coping responses, support experiences and needs, and the reconstruction of caregiving meaning and identity. Before formal data collection, three pilot interviews were conducted with caregivers who met the inclusion criteria to assess the clarity, relevance, and sensitivity of the interview questions. Data from the pilot interviews were not included in the final analysis. Minor revisions were made to the interview guide after discussion within the research team. The full interview guide is provided in Table 1.

**TABLE 1:** Interview guide.

● Could you describe the caregiving and rehabilitation-related responsibilities you mainly undertake as a family caregiver of a child with cerebral palsy?
● How has caregiving influenced your daily life, family role, work, or social relationships?
● How do you see yourself as a parent or family caregiver in the process of caring for your child? Has this understanding changed over time?
● In your interactions with relatives, neighbors, friends, schools, rehabilitation institutions, or public settings, have you experienced misunderstanding, staring, gossip, judgment, or unfair treatment toward your child, yourself, or your family? Could you give an example?
● How do you usually respond or cope when others ask about your child’s condition, or when your child receives attention, comments, or misunderstanding from others?
● In your child’s rehabilitation and daily care, have you ever felt that you must do everything possible, must not give up, or need to prove that you are a responsible caregiver? How have these feelings affected you?
● What people, events, or experiences have helped you adapt to your caregiving life and reinterpret your role as a caregiver? What support do you think healthcare professionals, rehabilitation institutions, communities, or society should provide for family caregivers of children with cerebral palsy?

Interviews were conducted in private and quiet settings at the study site to protect participants’ privacy and minimize interruptions. Before the interview, the interviewer explained the purpose of the study, the voluntary nature of participation, the approximate interview duration, audio-recording arrangements, confidentiality procedures, and the participants’ right to withdraw. Written informed consent was obtained before participation. After transcription, four participants were re-contacted by telephone to clarify ambiguous or insufficiently clear statements.

The interviews were conducted by two researchers (MY and ZZ), both of whom had received training in qualitative interviewing. One researcher led the interview using the semi-structured interview guide, while the other took field notes on participants’ emotional responses, pauses, tone, and other relevant non-verbal information. The interviewers had no prior relationship with the participants. During the interviews, the researchers used probing and clarifying questions to explore key issues in depth and to confirm important information. Particular attention was paid to participants’ emotional responses when discussing stigma, guilt, family judgment, or distressing caregiving experiences. If a participant showed signs of marked psychological distress, the interview could be paused or discontinued according to the participant’s preference. When additional support was needed, the hospital’s psychological nursing team was available to provide timely psychological support with the participant’s agreement.

Each interview lasted approximately 30–50 min. All interviews were audio-recorded with participants’ permission and transcribed verbatim. Field notes were reviewed together with the transcripts to support contextual understanding of the data.

### 2.4 Data analysis

Audio recordings were transcribed verbatim in Chinese within 24 h after each interview. To preserve the integrity of the data, the transcripts retained participants’ original wording as far as possible, including pauses, emphases, and emotional expressions where relevant. Field notes were reviewed together with the transcripts to support contextual understanding of participants’ accounts. All transcripts were anonymized before analysis and imported into NVivo 14 to facilitate data management, coding, retrieval, and comparison.

Data were analysed using inductive content analysis (Graneheim et al., 2017). This approach was selected rather than thematic analysis because the study aimed to organize caregivers’ accounts into practice-oriented descriptive categories and themes while remaining close to participants’ data, consistent with the qualitative descriptive design and study aim. Although the interview guide was informed by the study aim, relevant literature, expert consultation, and discussion within the research team, the analytic process was inductive: no predefined coding framework or theoretical categories were applied, and codes, categories, subthemes, and themes were developed from participants’ accounts. The analysis was conducted in Chinese to preserve the meaning of participants’ original expressions.

The analysis followed several iterative steps. First, the researchers repeatedly read the transcripts to gain an overall understanding of each participant’s account. Second, meaning units relevant to the study aim were identified and coded line by line. Third, codes with similar meanings or shared characteristics were compared and grouped into preliminary categories. Fourth, through repeated comparison and discussion, the categories were further refined, integrated, and named as subthemes and themes. Fifth, the developing thematic structure was checked against the original transcripts to ensure that the themes were grounded in the data and reflected both shared patterns and variation across participants.

Coding, categorization, and theme refinement were independently undertaken by three researchers (MY, CJ, and ZZ). When differences in coding or interpretation arose, the researchers returned to the original transcripts for repeated checking and resolved discrepancies through discussion within the research team until consensus was reached. Representative quotations were selected to illustrate each theme. After the analysis was completed in Chinese, MY and ZZ independently translated the themes, subthemes, and selected representative quotations into English and resolved discrepancies through discussion. MG, who had extensive overseas academic experience and strong English proficiency, reviewed the translations against the original Chinese text for semantic accuracy and preservation of culturally specific meanings. As an additional check, selected representative quotations were back-translated into Chinese by MY and compared with the original Chinese quotations before finalization.

### 2.5 Rigor and reflexivity

Trustworthiness was addressed through strategies related to credibility, dependability, confirmability, and transferability (Korstjens and Moser, 2018). Credibility was supported through purposive and maximum variation sampling, pilot interviews, open-ended semi-structured interviews, probing and clarification during interviews, and the use of field notes to supplement interview transcripts. Data collection and preliminary analysis proceeded iteratively, allowing emerging issues to be explored in subsequent interviews. Dependability was enhanced by following a consistent interview procedure and documenting the processes of data collection, transcription, coding, category development, and theme refinement. Confirmability was supported through independent coding by three researchers, repeated comparison of coding decisions, discussion of alternative interpretations, and checking the developing themes against the original transcripts. The research team included MY and ZZ, postgraduate nursing students with research interests in psychological nursing and prior qualitative interviewing experience; CJ, a clinically experienced nurse; and CX and MG, qualitative research experts. YW and BW assisted with participant recruitment and data collection arrangements. Recognizing that these professional backgrounds and assumptions could influence data collection and interpretation, the team engaged in regular reflexive discussions and considered alternative interpretations during analysis. Interpretations were repeatedly checked against the original Chinese transcripts to minimize the influence of any single professional perspective and ensure that the findings remained grounded in participants’ accounts. To support transferability, detailed information was provided about the research setting, participant selection, caregiver and child characteristics, and representative quotations, enabling readers to judge the relevance of the findings to other caregiving and rehabilitation contexts.

### 2.6 Ethics

Ethical approval was obtained from the Ethics Committee of Shandong First Medical University on 28 October 2024, before participant recruitment and data collection (Approval No. R202412220521). The approval covered the study procedures, including access to relevant medical-record information. The study was conducted in accordance with the ethical principles of the Declaration of Helsinki. Written informed consent was obtained from all participants before the interviews. If further professional psychological support was needed, the research team could, with the participant’s consent, facilitate referral to the psychological outpatient service at The First Affiliated Hospital of Shandong First Medical University. All data were anonymized and stored securely, with access limited to the research team.

## 3 Results

A total of 20 family caregivers shared their experiences of caring for children with cerebral palsy and managing stigma in everyday caregiving contexts. Participant and child characteristics are shown in Table 2. Data analysis generated four themes and fourteen subthemes. Together, these themes describe how family caregivers’ roles and expectations were disrupted and expanded after the child’s diagnosis, how stigma and social judgment were encountered in public and familiar social contexts, how caregivers managed stigma in everyday interactions, and how they reconstructed a valued caregiver identity through responsibility, support, and meaning-making. These changes in roles and expectations reshaped how caregivers understood themselves and their family responsibilities, providing the caregiving and identity context for their experiences of stigma and its management. The themes and subthemes are shown in Table 3. The analytical relationships among the four themes are illustrated in Figure 1. The arrows indicate conceptual relationships between the themes rather than a fixed temporal sequence or causal pathway.

**TABLE 2:** Demographic profile of the study participants (*n* = 20).

Number	Identity	Age	Education level	Work status	Monthly income	Residence	Care duration	Child age	Child sex	Classification
N1	Mother	34	Primary school	On leave	3000∼6000	Rural	4 years	6	M	Spastic type
N2	Father	27	Bachelor’s degree	Employed	>6000	Urban	3 months	3	F	Spastic type
N3	Mother	38	Junior college	Unemployed	3000∼6000	Urban	8 years	8	M	Spastic type
N4	Mother	40	Senior high school	Resigned	3000∼6000	Urban	8 years	12	M	Dyskinetic type
N5	Mother	37	Primary school	Unemployed	<3000	Rural	8 years	9	F	Spastic type
N6	Mother	32	Junior college	Resigned	3000∼6000	Urban	4 years	5	M	Spastic type
N7	Mother	33	Bachelor’s degree	Unemployed	3000∼6000	Urban	3 years	3	M	Spastic type
N8	Mother	40	Senior high school	Unemployed	<3000	Rural	7 years	9	M	Spastic type
N9	Mother	36	Junior college	Resigned	3000∼6000	Urban	4 years	6	M	Athetoid type
N10	Mother	28	Junior college	Unemployed	<3000	Rural	3 years	3	M	Spastic type
N11	Mother	35	Primary school	Unemployed	<3000	Rural	4 years	6	M	Spastic type
N12	Father	39	Bachelor’s degree	Employed	>6000	Urban	1 year	9	F	Spastic type
N13	Mother	45	Primary school	Unemployed	3000∼6000	Urban	9 years	9	F	Spastic type
N14	Mother	40	Primary school	Resigned	3000∼6000	Rural	1 year	1	M	Spastic type
N15	Father	40	Senior high school	Employed	3000∼6000	Urban	8 months	7	M	Spastic type
N16	Grandfather	56	Primary school	Retired	<3000	Rural	9 years	9	M	Spastic type
N17	Grandmother	78	Primary school	Retired	3000∼6000	Urban	12 years	12	M	Spastic type
N18	Father	33	Junior college	Employed	3000∼6000	Urban	9 months	1	M	Spastic type
N19	Mother	46	Bachelor’s degree	Employed	>6000	Urban	2 years	8	F	Spastic type
N20	Mother	39	Junior college	Employed	>6000	Urban	9 months	4	M	Spastic type

Care duration refers to the period during which the participant had served as the child’s primary family caregiver, rather than the total time since the child’s diagnosis. F, female; M, male.

**TABLE 3:** Overview of the themes and sub-themes.

Themes	Sub-themes
Becoming a caregiver: disrupted expectations and expanded family roles	Disrupted expectations of parenthood after diagnosis
	Moving from existing family roles into primary caregiving roles
	Caregiving displacing work, routines, and social life
Living under the gaze: stigma and social judgment in public and family encounters	Being exposed to staring, questioning, pity, and mockery in public spaces
	Blame and moral judgment within families and familiar communities
	Anticipated stigma and internalized affiliate stigma
Managing stigma in everyday interactions: concealment, disclosure, and resistance	Hiding, withdrawing, and controlling who knows
	Using ambiguous explanations to soften the label
	Naming cerebral palsy and reframing the child through rehabilitation and progress
	Confronting hurtful comments and asserting dignity
Rebuilding a valued caregiver identity through responsibility, support, and meaning-making	Continuing rehabilitation as a marker of responsible caregiving
	Small gains and the child’s responses as evidence that care matters
	Peer, workplace, professional, and informal support as resources for identity reconstruction
	Adjusting expectations while confronting an uncertain future

**FIGURE 1:**
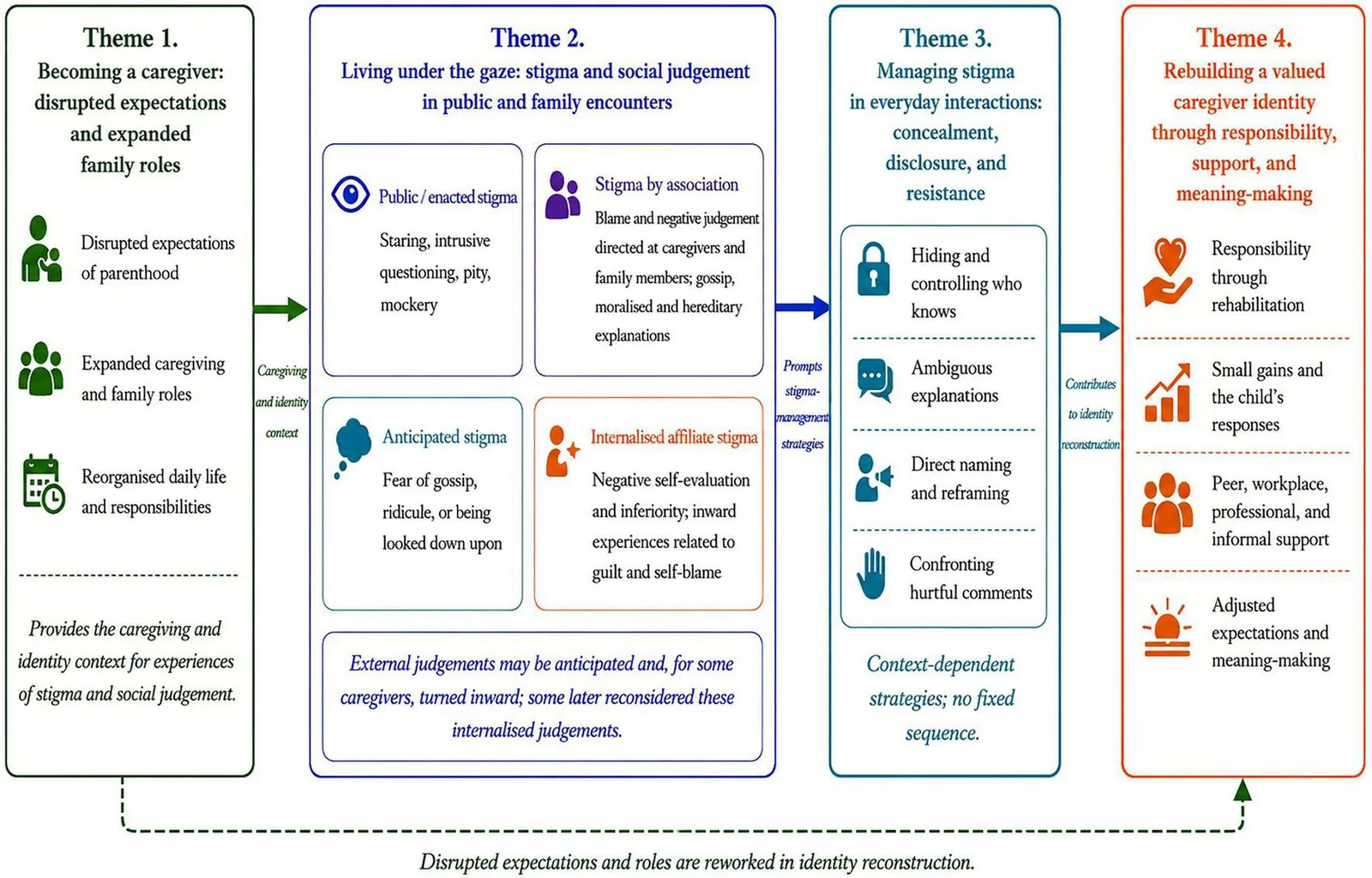
Conceptual diagram of stigma and caregiver identity reconstruction.

### 3.1 Becoming a caregiver: disrupted expectations and expanded family roles

This theme captures how caregivers’ previous expectations of parenthood and existing family roles were disrupted and expanded as the child’s diagnosis and rehabilitation needs became part of everyday life. Caregivers described changes in their expectations of parenthood, moving from previous family roles into primary caregiving roles, and reorganizing work, routines, and social relationships around care. These changes reshaped how caregivers understood themselves and their responsibilities within the family, providing an important identity context for their experiences of stigma and social judgment.

#### 3.1.1 Disrupted expectations of parenthood after diagnosis

Several participants described an early sense of rupture when the child’s development did not follow the trajectory they had previously expected. This rupture was not only about the child’s future, but also about how caregivers understood themselves as parents. Initial accounts were often marked by shock, fear, and difficulty imagining the future.

*“At first, I felt as if the sky had fallen. My child was so young and had already been diagnosed with cerebral palsy. I kept thinking, what will happen to me in the future?”*

*(N2)*

Furthermore, for some caregivers, comparisons with typically developing children made the gap between their previous expectations and their current caregiving reality particularly visible. This could be accompanied by feelings of parental inadequacy or failure as they confronted developmental differences they had not anticipated.

*“I felt like a failure. Other children could already run and jump, but my son could not even sit steadily. I feel I’m a complete failure.”*

*(N1)*

Moreover, the disruption extended beyond expectations for the child’s development. Most participants described how intensive caregiving disrupted the family life they had previously imagined. Their previous plans were interrupted, and everyday family life became increasingly centered on the child’s care. Changes in work and personal plans made this disruption particularly visible.

*“I quit my job eight years ago to take care of him full time, and I felt that my whole life was over.”*

*(N3)*

*“Heavy caregiving demands constantly disrupt my prior plans. The life we had imagined as parents has transformed entirely, and our entire family life has been disrupted.”*

*(N9)*

#### 3.1.2 Moving from existing family roles into primary caregiving roles

Caregiving also changed the boundaries of family roles. Participants described moving from roles that were previously centered on earning money, helping temporarily, or providing occasional support into more direct and sustained caregiving. This transition often began with concrete caregiving tasks rather than an explicit decision to become a primary caregiver. Through daily care, rehabilitation exercises, emotional support, and repeated decision-making, participants gradually came to recognize that their family role had changed.

For some participants, the previous division of family responsibilities became insufficient once rehabilitation and daily care entered ordinary life. Caregiving required them to move beyond financial or occasional support and become directly involved in bodily care, emotional support, and rehabilitation work.

*“I used to think that being a father meant earning money for the family and that the child’s care was his mother’s responsibility. Now it is different; I have to do it myself… A father is not just a cash machine. He also has to be a rehabilitation therapist, a nanny, and a psychologist.”*

*(N15)*

Role expansion became more pronounced when multiple responsibilities were concentrated in one caregiver. Participants described how everyday life shifted from previous personal routines toward care-centered routines, and how they had to learn forms of care they had not expected to perform.

*“Everything changed. I used to be able to play games and drink with friends, but now I go home after work to care for the child. My family role? I am both father and mother. I used to know nothing, but now I can change diapers faster than a nurse.”*

*(N18)*

For others, the transition into primary caregiving was gradual rather than planned. What began as temporary help could become a sustained caregiving responsibility when caregiving needs persisted and other family members were absent or unable to provide care.

*“I thought I was only helping for a while until his parents came back. Later, I realized they were not coming back, and I became the main person… He is my grandson. If I do not care for him, who will?”*

*(N16)*

#### 3.1.3 Caregiving displacing work, routines, and social life

As caregiving responsibilities expanded, participants described how work, daily routines, and social relationships were reorganized around the child’s care and rehabilitation. For some, this meant leaving work, reducing social contact, or negotiating between paid employment and daily rehabilitation tasks. Caregiving was therefore experienced not as a separate activity added to everyday life, but as something that reshaped the organization of daily living.

This reorganization was evident in the way paid work and caregiving responsibilities had to be negotiated around rehabilitation schedules. Participants described reducing overtime, avoiding work-related travel, leaving work on time, or taking leave for rehabilitation appointments.

*“My work has also been affected. I used to be able to work overtime and travel for work, but now I have to leave on time…. My family role? I am a single mother, the one who earns money, the one who provides care, and also the rehabilitation therapist. My social life is basically gone; when friends invite me to dinner, I usually refuse.”*

*(N1)*

For other participants, the reorganization of everyday life was described more broadly as a shift from previous personal routines to care-centered routines. Work, leisure, and social contact were all affected as daily life became increasingly organized around the child’s needs.

*“Work has also been affected because I often have to take leave for rehabilitation…. My social life is basically gone; when friends invite me, I do not go. Even if I go, I have to take the child with me, and others find it troublesome.”*

*(N12)*

### 3.2 Living under the gaze: stigma and social judgment in public and family encounters

Caregivers described stigma and social judgment as part of ordinary encounters in public places, families, and familiar communities. Stigma and social judgment were experienced not only through explicit insults, but also through staring, questions, pity, silence, gossip, and blame. In some situations, negative judgment extended from the child to the caregiver or wider family. Caregivers also anticipated negative reactions from others. In some cases, they internalized these judgments as feelings of inferiority or shame.

#### 3.2.1 Being exposed to staring, questioning, pity, and mockery in public spaces

Public spaces were described as difficult to enter because the child’s physical differences or behaviors could quickly attract attention. Some caregivers were hurt not only by overt ridicule, but also by looks, silence, or brief questions that marked the child as different. These encounters often made caregivers feel exposed even when others did not appear intentionally hostile.

*“When I took him to the mall, a child pointed at him and asked his mother, ‘Why is he like that?’ The mother quickly pulled the child away and looked back at me. That look was not malicious, but it made me feel that we were different.”*

*(N15)*

However, the attention participants described was not always openly hostile. Looks, silence, avoidance, or unfinished conversations could still make them feel exposed, because these reactions marked the child and family as different without necessarily expressing explicit hostility.

*“Once, when I was at the market carrying him, an old woman came up and looked at him, clicking her tongue with a ‘tsk-tsk’ sound. I burst into tears right there. Although she didn’t say anything, I could feel the disgust and pity that her reaction conveyed.”*

*(N7)*

*“Oh, when I took him to the hospital for rehabilitation, the doctor asked, ‘Where’s the child’s mother?’ I said, ‘Not here.’ The doctor didn’t say anything else, but a parent nearby gave me a look–that kind of look… as if I were pitiable.”*

*(N9)*

More direct questions or comments could lead participants to withdraw from the situation and carry the emotional impact afterward. In these moments, public attention was not only embarrassing, but also emotionally difficult to absorb.

*“Once a child asked, ‘Why does he walk like that?’ I quickly pulled my child away and cried after we got home. Whose child never gets ill? It is just that my child’s condition is visible.”*

*(N3)*

#### 3.2.2 Blame and moral judgment within families and familiar communities

In familiar communities, stigma was often expressed through gossip, blame, and moralized explanations of the child’s condition. In some accounts, blame and negative judgment were directed not only at the child but also at caregivers and other family members. Some caregivers in rural communities described how private family matters could quickly become the subject of public talk. In these settings, cerebral palsy was sometimes interpreted as a consequence of family wrongdoing, heredity, or poor family circumstances.

*“Rural folks have sharp tongues. One person actually said, ‘What’s the point of keeping a good-for-nothing like that?’ I felt like punching them. Even relatives told me, ‘Have another kid and dump this one on the grandparents.’ ”*

*(N10)*

*“They said ‘a child like that is just a burden to the whole family,’ and even my in-laws got pointed at when they went out. It felt like the whole family was tainted.”*

*(N11)*

Additionally, blame appeared within kinship and former marital relationships. In these situations, the child’s condition was sometimes attributed to family background or heredity, making participants feel that the family line itself was being judged.

*“The hardest part was that my ex-wife’s family kept saying, ‘There is something wrong with their family’s genes.’ When those words reached me, I was so angry that I wanted to fight.”*

*(N18)*

Compared with the brief encounters described in public spaces, these familiar-community judgments were harder for caregivers to escape. They were embedded in ongoing relationships and could repeatedly return through relatives, neighbors, or village gossip.

*“There was so much gossip in the village. People said behind my back, ‘The Chen family is raising a crippled child…’I was so angry that I could not eat for three days.”*

*(N16)*

#### 3.2.3 Anticipated stigma and internalized affiliate stigma

Some caregivers anticipated stigmatizing reactions from others even when no direct judgment had yet occurred. They worried that others might gossip about, ridicule, or look down on them because of the child’s condition.

“*At first, I did not dare to take him to the playground in the residential compound. I was afraid that other parents would talk about him.”*

*(N6)*

*“I was especially afraid of my colleagues finding out. I work in the city, and my workmates are all young people. I was terrified they would laugh at me for ‘having a stupid child.’ ”*

*(N12)*

By contrast, for some caregivers, derogatory comments about the child had already been encountered and were accompanied by feelings of inferiority. These external judgments became closely linked to how caregivers viewed themselves.

*“ ‘Her child is stupid.’ After I heard that, I could not eat for several days and felt inferior.”*

*(N1)*

*People often say unkind things about my child. I can’t help feeling inferior because of those words. How others see my child makes me feel bad about myself too.*

*(N6)*

Additionally, social comparison and uncertainty about the cause of the condition were accompanied by feelings of inferiority, guilt, and self-blame. Participants sometimes compared their child with typically developing children or looked back at pregnancy, family conflict, or caregiving decisions as possible reasons for the child’s condition.

*“When I see other people’s children who are so healthy, I wonder why my child has a disability. I always feel inferior, as though I’m not good enough.”*

*(N9)*

*“It is all my responsibility. I made him suffer so much. I really deserve to die.”*

*(N6)*

However, these negative self-evaluations and self-blaming interpretations were not uniformly maintained. Some participants described reconsidering earlier self-blame and rejecting the idea that other people’s words should determine how they viewed themselves or their children.

*“Later I came to understand that I could not stop loving my child just because of other people’s words.”*

*“If I look down on myself, my child will have even less hope.”*

*(N10)*

### 3.3 Managing stigma in everyday interactions: concealment, disclosure, and resistance

Across the interviews, caregivers described adjusting how they responded to stigma in different interactional contexts. Their strategies included concealing the diagnosis, using partial disclosure, naming cerebral palsy more directly, and openly responding to hurtful comments. These responses were not fixed, but varied according to whom caregivers were speaking to, the level of trust in the relationship, and the pressure created by the interaction.

#### 3.3.1 Hiding, withdrawing, and controlling who knows

For some participants, managing stigma meant limiting how far information about the child’s diagnosis could spread. In rural or closely connected communities, disclosure to relatives or neighbors was not seen as a single conversation, but as the point at which the child’s condition might become public talk and be retold by others.

*“I live in a rural village. If my relatives knew, the whole village would know, and the story would definitely become worse as it spread. I did not want that to happen.”*

*(N11)*

*“I am afraid that his grandmother would not be able to accept it. Right now, only his father and I know; the rest of the family do not know.”*

*(N14)*

Concealment could therefore protect the child and family from further exposure, but it also created discomfort for participants themselves. Some described feeling uneasy when they avoided the diagnosis or offered an explanation that did not fully match the child’s condition.

*“Once, a neighbor asked, ‘Why does your child always tilt their head?’ I casually said, ‘They are sleepy,’ and quickly walked away. Hiding like this made me feel very uncomfortable, as if I were a liar.”*

*(N2)*

Avoiding neighbors or acquaintances was another way of reducing situations in which participants had to explain the child’s condition repeatedly. This withdrawal was not only about avoiding questions, but also about avoiding disbelief, pity, and the feeling of being judged again.

*“Now when I meet neighbors or acquaintances, I would rather pretend not to see them or take another route. I do not want to explain everything from the beginning again, only to see that they still do not believe me and look at me with pity.”*

*(N7)*

*“I do not want to talk to them. When other people ask about my child, I feel that they are here to laugh at me.”*

*(N10)*

#### 3.3.2 Using ambiguous explanations to soften the label

Participants described using less direct terms when they were asked about the child’s condition but did not want to name cerebral palsy. These terms often focused on development or rehabilitation, such as saying that the child had “developmental problems” or was “doing rehabilitation.”

*“I used to avoid it completely, and even lied. Now I have started trying to say, ‘My child has some developmental problems and is doing rehabilitation.’ Although I still do not dare to say ‘cerebral palsy’ directly, this is already progress.”*

*(N2)*

Some participants described using everyday expressions that sounded less serious or were perceived as less stigmatizing than the diagnostic label. They used words such as “slow development” or “not very good legs” to answer others while avoiding the diagnostic label.

*“Before, when people asked, ‘How old is your child and she still can’t walk?’ I would explain, ‘She was premature, and her development is slow.’ ”*

*(N13)*

*“I once felt so ashamed that I would resort to saying ‘slow development.’ “*

*(N18)*

Participants also used explanations linked to the child’s current behavior or rehabilitation activity. When the child’s behavior attracted attention, this allowed them to give a simple explanation without turning the interaction into a discussion of the diagnosis.

*“Sometimes when he shouts and people look over, I just smile and say, ‘It’s okay. He is practicing making sounds.’ ”*

*(N15)*

#### 3.3.3 Naming cerebral palsy and reframing the child through rehabilitation and progress

Some participants described naming cerebral palsy more directly in some interactions, but they rarely presented the diagnosis on its own. When they used the term “cerebral palsy,” they often immediately linked it with treatment, rehabilitation, or the child’s progress.

*“In the past, I felt embarrassed and would just say, ‘His legs were not good.’ Now I am not afraid anymore. When people ask, I say, ‘It is cerebral palsy, and he is being treated. It has been nine years.’ ”*

*(N16)*

*“I used to say, ‘She is developing slowly.’ Now I say directly, ‘She has cerebral palsy, but she is making progress.’ I found that the more open I became, the more others respected me.”*

*(N19)*

Others described the diagnosis alongside the child’s abilities. The child was introduced as having cerebral palsy, but also as learning, practicing, or showing strengths in everyday life.

*“I used to say, ‘There is a problem with muscle tone,’ which people did not understand. Now I say directly, ‘It is cerebral palsy. He is eight years old and still learning to walk, but he is very smart and can recite Tang poems.’ ”*

*(N3)*

In some public interactions, caregivers used direct and simple explanations when the child’s difference attracted questions. These explanations combined the diagnosis or condition with what the child was learning to do.

*“In the past, I would speak vaguely and say, ‘His legs are not very good.’ Now I say directly, ‘He has cerebral palsy, but we are actively doing rehabilitation.’ The more clearly I say it, the less others make random guesses. Once, at the playground, a child asked, ‘Why doesn’t he run?’ I said, ‘He is learning slowly. Could you cheer for him?’ The child really cheered for him, and my son smiled.”*

*(N20)*

#### 3.3.4 Confronting hurtful comments and asserting dignity

Participants described responding more directly when comments were experienced as hurtful, stigmatizing, or beyond ordinary curiosity. These responses were not described as being used in every interaction, but appeared when participants felt that the child or family was being looked down upon.

*“If someone says something unpleasant, I directly reply, ‘Does your family’s child have no problems?’ ”*

*(N8)*

In some situations, direct responses were used to correct inaccurate assumptions about the child’s condition. This was particularly evident when others treated cerebral palsy as contagious or used words that participants experienced as humiliating.

*“I used to cry and say, ‘He is ill.’ Now I hold my head up and say, ‘It is cerebral palsy. It is not contagious. What do you want?’ The change is that I have gone from being weak to being tough.”*

*(N10)*

At other times, confrontation was directed at comments that interpreted disability-related behavior as poor discipline. Participants named cerebral palsy to challenge the explanation being placed on the child’s behavior.

*“Once she was shouting while sitting in her wheelchair, and someone nearby said, ‘Why is this child so disobedient?’ I became angry and said, ‘She has cerebral palsy. Do you not understand?’ ”*

*(N19)*

For some participants, confronting comments marked a shift from hiding, vague explanation, or pretending not to hear toward more open responses. They still described the child’s limitations, but also included what the child could do.

*“In the past, I would say, ‘He is physically weak,’ or pretend not to hear. Now I say directly, ‘It is cerebral palsy. He cannot walk, but he can smile and recognize people.’ If someone says something unpleasant, I push back: ‘If you have nothing to do, go pick up rubbish at the village gate.’ I no longer hide.”*

*(N4)*

In this way, direct confrontation was used selectively when participants felt that silence or vague explanation was no longer enough to protect the child from being misunderstood or diminished.

### 3.4 Rebuilding a valued caregiver identity through responsibility, support, and meaning-making

Caregivers described several experiences that helped them recognize value in their caregiving role. These included continuing rehabilitation despite fatigue or limited resources, noticing small changes in the child, receiving support from others, and adjusting expectations while facing an uncertain future.

#### 3.4.1 Continuing rehabilitation as a marker of responsible caregiving

Participants described rehabilitation as a routine that structured other parts of family life. It was not limited to scheduled therapy sessions, but extended into evenings, weekends, and repeated home practice, making caregiving responsibility visible through everyday time and effort.

*“The time after I come home from work is fixed for her. I first do language training, then fine motor training, and after dinner another hour of physical therapy. On weekends, I take her to the rehabilitation center for the whole day. I sleep only five hours a day, but I feel it is worth it.”*

*(N19)*

At the same time, the continuity of rehabilitation often took priority over caregivers’ own physical needs. Participants described rehabilitation as something that was difficult to interrupt, even when their own health or energy was limited.

*“I accompany my child to the rehabilitation center for her sessions every day. I wouldn’t dare miss a session even when I’m ill.”*

*(N2)*

When formal rehabilitation was constrained by cost, distance, or service access, caregivers described adapting rehabilitation into more accessible forms of daily care. They learned massage, exercises, or other practical skills themselves, and used local health workers’ advice to continue rehabilitation at home.

*“My family is poor, and my husband sends back just over two thousand yuan a month, so I have to spend carefully. But rehabilitation cannot stop, so I teach myself massage and exercises… I cannot give up. If I give up, then he really has no one.”*

*(N10)*

*“The rehabilitation center in town is too far away, and I can’t afford the travel costs. So I follow video tutorials, write down the movements, and do the rehabilitation massage for him every morning and evening. I think now I’m definitely no worse than those professional rehab centers.”*

*(N8)*

In these accounts, continuing rehabilitation was a practical way for caregivers to show responsibility, particularly when rehabilitation had to be sustained through limited time, money, health, and service access.

#### 3.4.2 Small gains and the child’s responses as evidence that care matters

Participants described judging the value of caregiving through incremental signs rather than through large developmental milestones. Within an uncertain rehabilitation process, even very small changes could make the effects of repeated care more visible.

*“The motivation comes from seeing a tiny bit of progress every day, such as holding a spoon for a few more seconds than before.”*

*(N1)*

*“Once, during a rehabilitation session, he suddenly stood by himself for three seconds, and everyone in the therapy room applauded. At that moment, I felt that all my efforts were worth it.”*

*(N3)*

At the same time, the child’s responses helped participants evaluate themselves differently as caregivers. Rather than relying only on external judgment, some participants described the child’s recognition or attachment as evidence that their care was meaningful.

*“Now I feel that I am not an incompetent mother. On the contrary, I am a very competent mother. My daughter smiles at me and calls me ‘Mum,’ and that is enough. I do not need to prove anything to others.”*

*(N19)*

For some participants, these responses were important because they made caregiving feel less one-sided. The child’s gestures of affection or concern allowed participants to see the relationship as reciprocal, even when the child continued to have significant care needs.

*“The most important moment was when my son kissed me on his own. I felt, ‘It was worth it.’ ”*

*(N11)*

*“Once, when I had a fever, my 3-year-old son used his not very flexible hand to bring me water. He spilled half of it, but I thought, he is so young and already knows how to care about me. What do I have to blame myself for?”*

*(N10)*

In this context, small changes and responses from the child became important because they made the effects of continued care visible in everyday life, even when broader developmental progress remained limited.

#### 3.4.3 Peer, workplace, professional, and informal support as resources for identity reconstruction

Support from others helped participants move away from interpreting caregiving only as a private failure or an individual burden. This shift was particularly evident when support came from people with similar caregiving experiences, because it allowed participants to place their situation within a shared caregiving struggle rather than seeing themselves as alone.

*“Later, I joined a cerebral palsy parents’ group, and a single father told me, ‘Think of him as your comrade-in-arms. The two of you are fighting a hard battle together.’ Those words woke me up. Now I feel that I am not a failure. I am just an ordinary father, and since this has happened, I have to carry on.”*

*(N18)*

This recognition was not limited to peer relationships. In settings where participants expected judgment, being believed or accommodated could make the caregiving role more visible and less shameful. Workplace support, for example, did not remove caregiving demands, but it changed how disclosure was experienced.

*“I cried while telling my supervisor the truth. My supervisor did not laugh at me and even helped adjust my working hours.”*

*(N2)*

Professional and educational support contributed in a different way by giving participants language to reinterpret what daily care meant. Rather than understanding their role only through illness, treatment, or burden, some participants began to describe caregiving as parenting, teaching, and supporting the child’s development.

*“A special education teacher once told me, ‘You are not caring for a patient; you are raising a child.’ Those words suddenly made things clear to me.”*

*(N3)*

Professional recognition also helped participants attach value to repetitive care work that could otherwise feel invisible. When therapists linked home-based practice with the child’s progress, daily routines were easier to understand as meaningful caregiving rather than as endless tasks.

*“A rehabilitation therapist told me that my child’s progress depended on the half-hour I did every day. So I gritted my teeth and kept going.”*

*(N15)*

Finally, at the everyday level, informal help from family members and friends made this reconstructed role more sustainable. It did not necessarily change the diagnosis or remove the demands of care, but it gave caregivers moments of relief and helped them feel less alone.

*“I am most grateful to my mother-in-law. She cared for him for more than three years and did it very well. I also have a close friend who comes every week to help for half a day, so I can go to the supermarket or get some sleep.”*

*(N20)*

Together, these forms of support did more than provide emotional comfort or practical assistance. They helped participants see caregiving as shared, recognized, and meaningful work, rather than only as a source of failure, shame, or isolation.

#### 3.4.4 Adjusting expectations while confronting an uncertain future

Participants described differences between their earlier expectations and how they now thought about the future. Earlier hopes were often linked to visible recovery, whereas some participants now described their hopes in smaller and more immediate terms. This did not mean that hope had disappeared; rather, it was tied to what could still be maintained in everyday family life.

*“I used to dream that he would suddenly stand up and run overnight. Now I feel that as long as he is alive and stays with us, it is already a blessing. The greatest happiness is that the three of us can be together.”*

*(N14)*

*“Now I feel that I am his only light. I do not expect him to become very successful in the future. If he can live and smile at me, I will be satisfied.”*

*(N10)*

However, this adjustment did not make the future easier to imagine. Participants’ uncertainty was closely connected to the fact that the child’s daily life still depended on their own presence, physical strength, and continued ability to provide care.

*“If one day something happens to me and I leave before my child, how will my child live? I really do not dare to think further.”*

*(N6)*

This concern became more concrete when participants thought about whether the care they were providing now could continue as both they and the child grew older. The future was therefore not only a matter of the child’s condition, but also of whether everyday care could still be carried out.

*“There is no suitable wheelchair or transport. I have to carry him to rehabilitation. Now he is still small, so I can carry him. What will I do when I am old?”*

*(N3)*

In the absence of a secure long-term arrangement, some participants described narrowing their attention to the time they could still spend with the child. Their accounts did not offer certainty about the future, but stayed close to what could still be done in the present.

*“I am 78 now, and the longer I care for him, the more afraid I become. I am afraid that no one will care for him after I am gone. Now I just take it one day at a time. If I can stay with him for one more day, then I will. I am not great; I simply have no other choice.”*

*(N17)*

## 4 Discussion

This qualitative study provides an in-depth account of how family caregivers of children with cerebral palsy experience and manage stigma-related social encounters and reconstruct a valued caregiver identity in the Chinese context. Overall, our findings showed that disruptions to previous expectations of parenthood and family roles provided an important caregiving and identity context for caregivers’ experiences of stigma and social judgment. These experiences included public/enacted stigma, stigma by association, anticipated stigma and, in some accounts, internalized affiliate stigma. Caregivers responded to these experiences through a range of stigma management strategies, including concealment, ambiguous explanation, direct naming of cerebral palsy, and resistance to hurtful comments. Furthermore, our findings highlight the close relationship between stigma management and caregiver identity reconstruction, showing how caregivers sought to protect the child’s dignity while recognizing value in their own caregiving role.

Our study found that caregiver identity reconstruction was closely tied to everyday caregiving responsibility and continued rehabilitation work. A qualitative synthesis by Smith and Blamires (2022) showed that mothers of children with cerebral palsy often reorganized their lives around caregiving, made substantial sacrifices, and experienced physical, emotional, financial, and social challenges. Nova-Díaz et al. (2025) further demonstrated that the economic and social burden of pediatric cerebral palsy was largely driven by caregiving and non-medical costs, including productivity losses among informal caregivers. These findings resonate with our participants’ accounts of disrupted work, reduced social participation, and daily life being reorganized around rehabilitation. However, our study further adds that these responsibilities were not described only as sources of strain. For some caregivers, continuing rehabilitation, learning home-based rehabilitation strategies, and noticing small changes in the child became concrete ways to describe what it meant to remain responsible and to “not give up” on the child. This interpretation is consistent with qualitative studies reporting that caregivers of children with cerebral palsy may also describe hopeful caregiving, purpose, resilience, pride, and strengthened relationships despite substantial caregiving demands (Lewis et al., 2025; Maronga-Feshete et al., 2024). These findings suggest that daily rehabilitation work should not be understood only as a source of caregiving burden; for some caregivers, it was also a context in which responsibility, competence, and value in the caregiving role were affirmed.

Beyond the reorganization of everyday caregiving, participants’ accounts drew attention to the social settings in which stigma and social judgment were encountered. Previous research has shown that parents of individuals with cerebral palsy may experience affiliate stigma in relation to caregiving stress and quality of life (Chu et al., 2023), while qualitative evidence has reported guilt, cultural blame, social stigma, marginalization, discrimination, social exclusion, and negative community perceptions among caregivers of children with cerebral palsy (Smith and Blamires, 2022; Mwinbam et al., 2023; Kisinna et al., 2025). Our study adds further nuance by showing that these stigma experiences occurred across different relational settings. Specifically, the child’s visible differences in movement, behavior, or communication exposed caregivers to public/enacted stigma through staring, intrusive questioning, pity, and mockery in public spaces. Within familiar social networks, including relatives, neighbors, village communities, and kinship or marital relationships, stigma by association was evident when blame, hereditary explanations, gossip, and moral judgment were directed toward caregivers or the wider family. These familiar-network encounters appeared especially difficult for caregivers to distance themselves from because they were embedded in ongoing relationships rather than brief public interactions. For some participants from rural communities, blame and gossip circulated through relatives, neighbors, and village networks. This finding may be understood in the Chinese cultural context, where concerns about family reputation, loss of face, shame, and social rejection can shape families’ experiences of disability-related stigma (Holroyd, 2003; Chiu et al., 2013; Huang et al., 2020; Ng and Ng, 2022). In such closely connected social networks, negative judgments about the child may extend to the wider family, making private caregiving experiences difficult to separate from broader social evaluation. This is consistent with Luetke Lanfer et al. (2025) who suggest that stigma may extend from the child to the caregiver through repeated social judgment, blame, and exclusion. Our findings further showed that some caregivers anticipated such negative reactions before they occurred, whereas in other accounts external judgments were internalized as inferiority or negative self-evaluation. This distinction situates internalized affiliate stigma within a broader relational context of public/enacted stigma, stigma by association, and anticipated stigma, while clarifying that these related forms of stigma should not be treated as interchangeable.

Practical communication support for caregivers was indicated by participants’ accounts to be an important condition for managing stigma in everyday encounters, facilitating clearer explanations of cerebral palsy, more confident responses to questions, and more controlled disclosure of the child’s condition. However, participants’ accounts also suggested several gaps that made these interactions difficult. These included uncertainty about whether to disclose the diagnosis, limited access to simple and non-stigmatizing ways of explaining cerebral palsy, and insufficient preparation for responding to intrusive questions, pitying comments, or moral judgments from others. Rather than following a fixed sequence, caregivers used concealment, vague explanations, direct naming, or confrontation according to the relationship and perceived risk of further exposure (Mateo-Arriero et al., 2025a,b). Our finding aligns with Lanfer et al.’ studies showing that caregivers of children with cerebral palsy or other disabilities experience stigma through blame, avoidance, social exclusion, and community misconceptions, and may respond through avoidance, discounting, acceptance, reaction (Dako-Gyeke et al., 2021), withdrawal, coping, or resistance (Luetke Lanfer et al., 2025). While these responses to stigma have been documented in the literature (Luetke Lanfer et al., 2025; Dako-Gyeke et al., 2021; Ng and Ng, 2022; Tekola et al., 2020), our study provides more specific insight into the role of communication in stigma management among caregivers of children with cerebral palsy. In particular, our findings show that caregivers were not only attempting to reduce their own embarrassment or distress, but were also actively shaping the social meaning of the child’s condition by pairing the diagnosis with rehabilitation, progress, and the child’s abilities.

In parallel, participants’ accounts also pointed to similar knowledge and communication gaps among people surrounding the family, including relatives, neighbors, other parents, teachers, and community members. These included limited recognition that cerebral palsy is a neurodevelopmental condition rather than a contagious disease or a result of family fault, insufficient understanding that some behaviors may be disability-related rather than intentional misbehavior, and limited awareness that rehabilitation progress and the child’s abilities should be recognized alongside impairment. Participants perceived these gaps as barriers to more inclusive social interaction, because they repeatedly placed caregivers in the position of having to explain, defend, or reframe the child’s condition. Research by Zuurmond et al. (2018) demonstrated that a participatory community-based parent training programme for caregivers of children with cerebral palsy, delivered through local support groups and emphasizing caregiver empowerment, improved caregiver quality of life, knowledge, and confidence in caring for the child. A related support programme for caregivers of children with disabilities also highlighted the value of participatory training and local support groups for caregiver wellbeing (Zuurmond et al., 2019). These previous findings, together with recent disability stigma-reduction guidance emphasizing multi-level work with caregivers, persons with disabilities, service providers, healthcare workers, education professionals, and community members rather than one-off awareness activities (Smythe et al., 2020; Rao et al., 2019), offer valuable insight for developing similar initiatives to address the communication and knowledge gaps identified in our study, and to further mitigate public misconceptions and societal prejudice toward cerebral palsy. Besides, we believe that, within the context of cerebral palsy care in China, these initiatives could be translated into more practical communication support and stigma-sensitive education. Importantly, our findings suggest that family-centered, stigma-sensitive care is necessary but may not be sufficient on its own, because stigma-related experiences arose across individual, family, community, and service contexts. For example, healthcare professionals and rehabilitation providers could help families develop accessible, understandable, and non-stigmatizing explanations of cerebral palsy; distinguish the child’s disability-related behaviors from misconceptions such as being “disobedient,” “contagious,” or the result of family fault; and prepare responses to intrusive questions, pitying comments, or erroneous attributions from others. Within families, brief discussions could be used to correct misconceptions about the causes of cerebral palsy, reduce blame directed toward the primary caregiver, and agree on how the child’s condition is explained to relatives or others. Meanwhile, schools and community services could provide stigma-sensitive education that presents cerebral palsy as a neurodevelopmental condition while emphasizing children’s abilities, participation, and rehabilitation progress. At the health-service level, rehabilitation and nursing services could incorporate routine attention to stigma-related concerns into follow-up care and establish clear pathways to peer and psychological support when additional help is needed. By reducing misconceptions, blame, and exclusion in everyday social settings, such efforts may help alleviate caregivers’ stigma-related distress and psychological distress. In this way, support would not only target caregivers’ psychological adjustment, but also improve the social communication environment in which caregiving takes place. These approaches may help shift part of the burden of managing stigma away from individual families and toward more inclusive care, education, and community settings, thereby providing a more stable social basis for caregiver identity reconstruction.

Finally, our study also identified that repeated affirmation of caregivers’ value was central to the reconstruction of a valued caregiver identity. In this process, children’s small rehabilitation gains and emotional responses, caregivers’ continued involvement in daily rehabilitation, and supportive interactions with peers, professionals, and family members were viewed as key enablers that helped caregivers reinterpret themselves as responsible and meaningful caregivers. Furthermore, participants’ accounts suggested that these sources of affirmation were particularly important because cerebral palsy diagnosis, caregiving burden, public stigma, and family judgment had disrupted their previous expectations of parenthood. And, for some caregivers, these stigma-related experiences were associated with feelings of perceived failure, guilt, or being negatively evaluated by others. Participants did not describe a valued caregiver identity as resting on acceptance alone; rather, they linked it to recurring sources of affirmation that their care still mattered, even when the child’s future remained uncertain. Existing literature has made visible the long-term conditions in which caregivers of children with cerebral palsy carry concentrated responsibility, experience isolation and exhaustion, face restrictions in everyday and social life, and live with uncertainty about the future (Ni et al., 2022; Dlamini et al., 2023; Smith and Blamires, 2022; Liu et al., 2023). They help explain the circumstances under which caregivers’ previous expectations of parenthood and self-understanding may become strained. However, less attention has been paid to how caregivers rebuild a valued caregiver identity and sense of self within these complex caregiving conditions. In this light, our findings provide more specific insight into how repeated affirmation may support this reconstruction process, rather than merely offering temporary comfort. Situating this finding within previous research also suggests that positive caregiving experiences are not necessarily separate from caregiving difficulty, but often emerge within it. Maronga-Feshete et al. (2024) reported that caregivers of children with cerebral palsy experienced financial, psychological, social, and physical challenges, while also describing increased resilience, strengthened relationships, and pride in their children. Lewis et al. (2025) similarly found that caregivers could identify hope, purpose, connection with the child, and relationship-specific support while living with emotional, physical, social, and financial demands and difficulty. These studies help show that the positive meanings described by participants in our study should not be interpreted as a denial of caregiving burden or as the opposite of hardship. What our findings add is that such experiences may participate in identity reconstruction itself, helping caregivers reinterpret continued caregiving as meaningful and valuable rather than remaining only positive moments alongside burden.

This understanding also suggests that supporting a valued caregiver identity requires attention to the service and relational contexts in which caregiving takes place (King and Chiarello, 2014; Cook et al., 2022; Akhbari Ziegler and Hadders-Algra, 2020). Poojari et al. (2022) showed that caregivers’ expectations of family-centered care extended beyond the child’s treatment itself, including information about the child’s progress and prognosis, community resources, empowerment, and support groups. Melak et al. (2025) also identified financial, informational, emotional, and physical support needs among caregivers of children with cerebral palsy, and called for greater attention to positive caregiving outcomes and caregivers’ inherent resources to inform strength-based interventions. Viewed from this perspective, such support may contribute not only to reducing caregiving burden, but also to sustaining a valued caregiver identity. Rehabilitation and nursing services should therefore not focus solely on children’s functional training, but also help caregivers recognize the significance of small rehabilitation gains, provide consistent feedback on the child’s progress, facilitate access to peer and family support, and discuss future care arrangements earlier. This suggests that future research is needed to clarify how family-centered, strength-based, and future-oriented support can help caregivers maintain a valued caregiver identity over time.

## 5 Strengths and limitations

This study has several strengths. First, it extends existing research on family caregivers of children with cerebral palsy beyond caregiving burden and support needs by examining how different forms of stigma were encountered and managed in everyday interactions and how caregivers reconstructed a valued caregiver identity. This focus allowed us to consider not only the negative consequences of stigma, but also the ways in which caregivers protected the child’s dignity, negotiated disclosure, and sought meaning in caregiving. Second, the qualitative descriptive design was appropriate for staying close to caregivers’ own accounts while generating findings with practical relevance for rehabilitation, nursing, family support, and community-based services. Third, purposive sampling sought variation across caregiver and child characteristics, helping to capture a range of caregiving experiences across family contexts.

However, several limitations should also be acknowledged. First, participants were recruited from a single tertiary hospital, most were mothers, and most children had spastic cerebral palsy, which may limit transferability to other caregiver groups and care settings. Standardized functional classification data, including the GMFCS, CFCS, and MACS, were not collected, limiting our ability to examine whether caregivers’ experiences differed according to the child’s motor, communication, or manual functional level. Second, the study focused on caregivers’ accounts and did not include the perspectives of children with cerebral palsy, healthcare professionals, rehabilitation providers, teachers, or community members. Therefore, findings related to public encounters, family judgment, professional support, and community responses should be understood primarily from caregivers’ perspectives. Third, the data were collected through retrospective interviews at one point in time, which may have limited our ability to capture how stigma management and caregiver identity reconstruction change across the caregiving trajectory. Participants’ accounts may also have been influenced by recall, emotional state, or social desirability. Finally, because the interviews were conducted in Chinese and selected quotations were translated for publication, some culturally embedded meanings and emotional nuances may not have been fully conveyed in English. Future research could use longitudinal, multi-site, and multi-perspective designs incorporating standardized functional classifications to further examine how stigma-related experiences and caregiver identity reconstruction vary across contexts and over time.

## 6 Conclusion

Our study provides valuable insight into how family caregivers of children with cerebral palsy in China experience and manage stigma-related social encounters and reconstruct a valued caregiver identity. Disruptions to previous expectations of parenthood and family roles provided an important context for these experiences, which included public/enacted stigma, stigma by association, anticipated stigma and, in some accounts, internalized affiliate stigma. Caregivers managed these experiences through context-dependent concealment, disclosure, explanation, and resistance. The reconstruction of a valued caregiver identity was supported by continued caregiving, children’s small rehabilitation gains and emotional responses, and recognition from supportive relationships, despite persistent uncertainty about the child’s future. These findings suggest that family-centered, stigma-sensitive care should go beyond children’s functional needs and caregivers’ individual adjustment. Support should also address stigma-related challenges within families, communities, and service settings. At the same time, it should strengthen the sources of support that help caregivers recognize their value and sustain a valued caregiver identity.

## Data Availability

Due to the sensitive nature of the interview data and participants’ confidentiality, the full transcripts are not publicly available. All data were anonymized and stored securely, with access limited to the research team. Inquiries about the data can be directed to the corresponding author/s.
